# User expectations and experiences of an assistive robotic arm in amyotrophic lateral sclerosis: a multicenter observational study

**DOI:** 10.1186/s42466-024-00342-3

**Published:** 2024-08-23

**Authors:** Susanne Spittel, Thomas Meyer, Ute Weyen, Torsten Grehl, Patrick Weydt, Robert Steinbach, Susanne Petri, Petra Baum, Moritz Metelmann, Anne-Dorte Sperfeld, Dagmar Kettemann, Jenny Norden, Annekathrin Rödiger, Benjamin Ilse, Julian Grosskreutz, Barbara Hildebrandt, Bertram Walter, Christoph Münch, André Maier

**Affiliations:** 1 Department of Neurology, Center for ALS and Other Motor Neuron Disorders, Charité – Universitätsmedizin Berlin, Augustenburger Platz 1, 13353 Berlin, Germany; 2 Ambulanzpartner Soziotechnologie APST GmbH, Berlin, Germany; 3 Department of Neurology, Center for ALS and Other Motor Neuron Disorders, Berufsgenossenschaftliches Universitätsklinikum Bergmannsheil, Bochum, Germany; 4 Department of Neurology, Center for ALS and Other Motor Neuron Disorders, Alfried Krupp Krankenhaus, Essen, Germany; 5 Department for Neuromusclar Disorders, Bonn University, Bonn, Germany; 6 Department of Neurology, Jena University Hospital, Jena, Germany; 7 Department of Neurology, Hannover Medical School, Hannover, Germany; 8 Department of Neurology, Universitätsklinikum Leipzig, Leipzig, Germany; 9 Department of Neurology, Sächsisches Krankenhaus Altscherbitz, Altscherbitz, Germany; 10 Department of Neurology, Universitätsmedizin Schleswig-Holstein, Campus Lübeck, Lübeck, Germany

**Keywords:** Amyotrophic lateral sclerosis, Motor neuron disease, Assistive technology devices, Assistive robotic arm, Platform, Case management

## Abstract

**Objective:**

Robotic arms are innovative assistive devices for ALS patients with progressive motor deficits of arms and hands. The objective was to explore the patients´ expectations towards a robotic arm system and to assess the actual experiences after the provision of the device.

**Methods:**

A prospective observational study was conducted at 9 ALS centers in Germany. ALS-related functional deficits were assessed using the ALS-Functional Rating Scale-revised (ALSFRS-R). Motor deficit of the upper limbs was determined using a subscore of three arm-related items of the ALSFRS-R (items 4–6; range 0–12 points). User expectations before provision (expectation group, n = 85) and user experiences after provision (experience group, n = 14) with the device (JACO Assistive Robotic Device, Kinova, Boisbriand, QC, Canada) were assessed.

**Results:**

In the total cohort, mean ALSFRS-R subscore for arm function was 1.7 (SD: 2.0, 0–9) demonstrating a severe functional deficit of the upper limbs. In the expectation group (n = 85), the following use cases of the robotic arm have been prioritized: handling objects (89%), close-body movements (88%), pressing buttons (87%), serving drinks (86%), and opening cabinets and doors (85%). In the experience group (n = 14), handling objects (79%), serving drinks (79%), near-body movements (71%), pushing buttons (71%), serving food (64%), and opening doors (64%) were the most frequent used cases. Most patients used the device daily (71.4%, n = 10), and 28.6% (n = 4) several times a week. All patients of the experience group found the device helpful, felt safe while using the device, and were satisfied with its reliability. NPS of the assistive robotic arm revealed 64% "promoters" (strong recommendation), 29% "indifferents" (uncertain recommendation) and 7% "detractors" (no recommendation). Total NPS was + 57 demonstrating strong patient satisfaction.

**Conclusions:**

Initiation of procurement with a robotic assistive arm was confined to patients with severe functional deficit of the upper limbs. User experience underlined the wide spectrum of use cases of assistive robotic arms in ALS. The positive user experience together with high satisfaction underscore that robotic arm systems serve as a valuable treatment option in ALS patients with severe motor deficits of the arms.

**Supplementary Information:**

The online version contains supplementary material available at 10.1186/s42466-024-00342-3.

## Introduction

Amyotrophic lateral sclerosis (ALS) is a fatal neurodegenerative disease characterized by a progressive loss of muscle and paresis of the extremities [1]. In the course of the disease, people with ALS suffer progressive paresis of the arms and hands. Both vital and apparently basic and everyday motor functions are severely affected. This results in the loss of manual functions and severe limitations in the patient's motor autonomy [2] and implies a high burden for the patient and the caregiver [3]. Assistive technology devices (ATD) play an important role in ALS care [4–7]. As a very recent device, robotic arms have been available for several years, being deficit-oriented ATD that can compensate for the loss of function caused by the disease [8]. A robotic arm enables the patient to perform elementary manual actions independently, including grasping and handling [8]. This can increase independence in the activities of daily living and motor self-determination. As people with ALS display high technological commitment, robotic arms are assumed to be an innovative ATD that promotes independence [2, 9].

Acceptance is reflected by increased users' willingness to use a robotic arm as the functionality of their own arms decreases [2]. In Germany, procurement of a robotic arm at the expense of health insurance is based on individual cost coverage procedures. Currently, there is little systematic data available on this treatment option, such as user expectations and experience in the use of robotic arms for ALS. The aims of this study were (i) to investigate the functional deficits leading to robotic arm procurement, (ii) to explore the treatment expectation, (iii) to assess the frequency of use of the robotic arm, (iv) to analyze the user experience and (v) to obtain the recommendation rate.

## Methods

### Study design

The observational study was conducted as a prospective, multicenter, cohort study. The investigation was reported according to the STROBE criteria [10, 11].

### Participants

The following inclusion criteria were defined for participation in the patient survey: (1) diagnosis of ALS according to the revised El Escorial criteria [12], (2) participation in a case management program for ALS, (3) consent to electronic data capture using the case management and research platform named “Ambulanzpartner”, (4) initiation or completion of procurement with a JACO Assistive Robotic Device (Kinova, Boisbriand, QC, Canada), (5) consent to observational registry study.

### Setting

#### Indicating the robotic arm and case management

The cohort observed encompassed patients who had received an indication for a robotic arm at 9 multidisciplinary ALS outpatient clinics in Germany over a 24-month observation period. The centers contributed to the multi-center case management and research platform. The medical indication for the provision with a robotic arm and the recruitment to the platform was ascertained by ALS-trained neurologists and encompassed the primary diagnosis of an arm paresis, which legitimated the robotic arm provision. Patient’s ability to operate and control the robotic arm was additionally required. Once these criteria were met, the patient was referred to a case manager specialized in ATD provision in ALS, who matched the robotic arm requirements with the device provider. The suitable service provider carried out a trial of the ATD together with the patient. As part of this trial, a video is usually produced that demonstrates feasibility and benefit to the health insurance company. In Germany, the coverage of costs of this device by health insurance companies depends on the results of the trial. The patient will only be provided after a successful appraisal process initiated by the health insurance company.

#### Data collection

Data collection took place during specialized medical consultations in ALS outpatient clinics. In the baseline survey data were collected from patients at time of indication where user expectations before provision were assessed. In the follow-up survey user experience were collected from patients who have used the robotic arm for at least three months.

The data about the procurement process and the causes of the failed provision were collected using the defined software components of the Ambulanzpartner (APST, https://www.ambulanzpartner.de/) platform [13]. The APST portal consisted of an electronic health record and a digital management platform, which has been described elsewhere [4, 14–17]. The platform links all participating ALS centers, case managers, and providers of the device and provided a multi-step workflow for the provision of ATD.

### Protocol approvals and registrations

The study protocol was approved by the Medical Ethics Committee of Charité – Universitätsmedizin Berlin, Germany under number EA1/219/15. A signed patient information and informed consent form was obtained from all the participating patients. The observational registry study has been registered at the German Clinical Trials Register (https://www.drks.de/DRKS00031710) and clinicaltrials.gov platform (NCT05852418).

### Variables

#### Demographic and clinical characteristics

The following demographic and clinical characteristics were collected: age, sex, time since onset of symptoms, and disease progression rate. An overview of the participants’ demographic and clinical characteristics is given in Table 1.

**Table 1:** Demographic and clinical characteristics of participants

Characteristics	Classification	Total cohort, n = 85	Provided with robotic arm, n = 32	Not provided with robotic arm, n = 53	*p*-value
Sex	Female, % (n)	30.6 (26)	25.0 (8)	34.0 (18)	0.268
	Male, % (n)	69.4 (59)	75.0 (24)	66.0 (35)	
Age	At onset, years, mean (SD, R)	52.5 (11.3, 26.4–81.3)	50.6 (10.5, 34.3–72.4)	53.7 (11.7, 26.4–81.3)	0.264
	At time of indication of robotic arm, years, mean (SD, R)	56.3 (10.7, 29.8–84.5)	53.1 (11.7, 22.2–75.3)	57.6 (10.7, 29.7–84.5)	0.140
	At time of provision or not-provision of the robotic arm, years, mean (SD, R)	54.7 (10.5, 36.8–75.9)	53.7 (11.8, 22.6–75.9)	57.8 (10.9, 30.6–83.9)	0.108
Disease duration	Years, mean (SD, R)	4.8 (4.0, 0.8–20.9)	5.2 (4.1, 0,8–17.5)	4.6 (4.0, 0.8–20.9)	0.496
Disease progression rate	Mean (SD, R)	0.64 (0.9, 0.1–5.9)	0.74 (1.0, 0.2–5.7)	0.58 (0.8, 0.1–5.9)	0.459
ALSFRS-R score (max. 48)	At time of indication, mean (SD, R)	23.8 (7.8, 7–40)	23.6 (7.8, 9–38)	23.8 (7.9, 7.0–40.0)	0.843
ALSFRS-R sub-score of the upper extremities^a^	At time of indication, mean (SD, R)	1.7 (2.0, 0–9)	1.5 (1.8, 0–6)	1.7 (2.2, 0–9)	0.653
Functional impairment of the upper extremities^a^	At time of indication, total, yes, % (n)	100 (85)	100 (32)	100 (53)	n/a
Severity of functional impairment of the upper extremities^a^	No limitation of arm function, % (n)	0 (0)	0 (0)	0 (0)	n/a
	Slight limitation of arm function, % (n)	1.2 (1)	0 (0)	1.2 (1)	
	Moderate limitation of arm function, % (n)	14.1 (12)	12.5 (4)	15.1 (8)	
	Severe limitation of arm function, % (n)	84.7 (72)	87.5 (28)	83.0 (44)	

^a^Severity of functional impairment of the upper extremities was determined by the sum of item 4 (handwriting), item 5 (cutting food and handling cutlery/feeding tube and utensils) and item 6 (dressing and personal hygiene) of the ALSFRS-R (12 possible points). The deficit of arm function was classified in the following four gradings: severe limitation of arm function (0–3 points), moderate limitation of arm function (4–7 points), slight limitation of arm function (8–11 points), and no limitation of arm function (12 points)

Abbreviations: n = number of participants; SD = standard deviation; R = range; ALSFRS-R = Amyotrophic Lateral Sclerosis Functional Rating Scale Revised, n/a = not applicable

#### Functional deficits by ALSFRS-R

Motor functional deficit was assessed by the ALS-Functional Rating Scale revised (ALSFRS-R, 48 possible points) [18–20]. ALS progression rate was calculated by loss of ALSFRS points per month since symptom onset. The functional deficit of the upper extremities was determined by the sum of item 4 (handwriting), item 5 (cutting food and handling cutlery/feeding tube and utensils) and item 6 (dressing and personal hygiene) of the ALSFRS-R (12 possible points). The deficit of arm function was classified in the following four gradings: severe functional deficit (0–3 points), moderate functional deficit (4–7 points), slight functional deficit (8–11 points), and no functional deficit (12 points).

#### User expectations

**Fig. 1:**
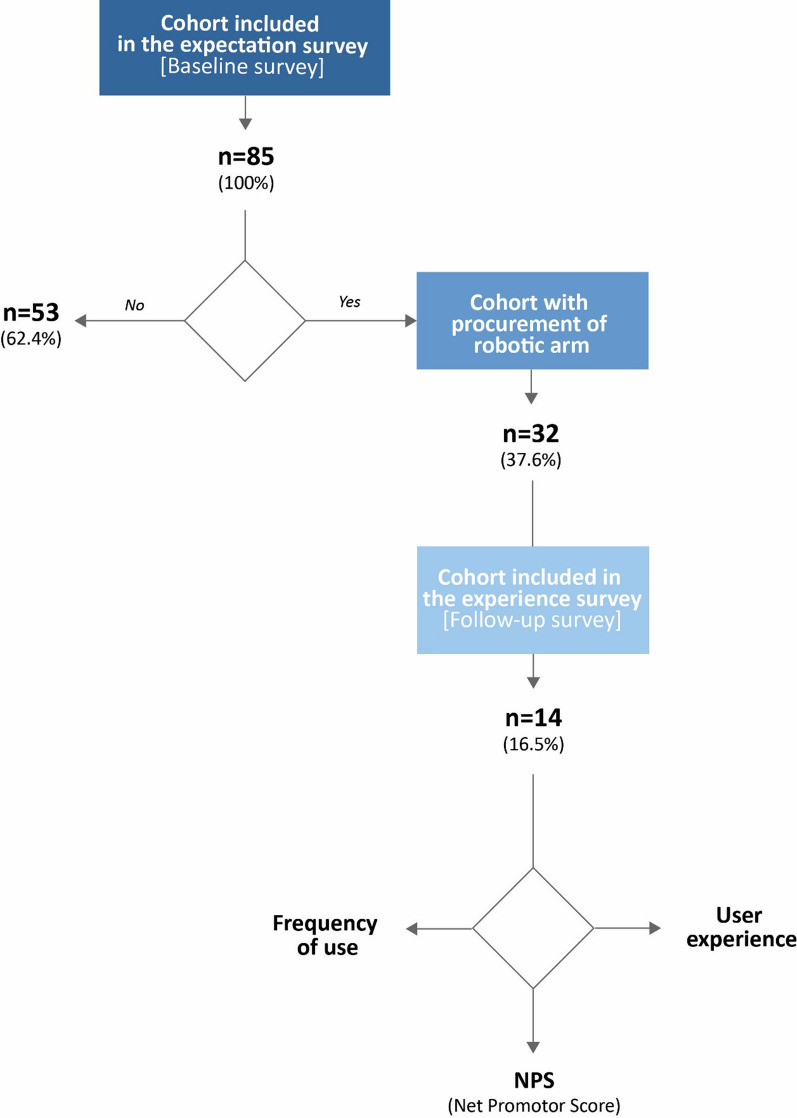
Sample characteristics. A total number of ALS patients at the study site fulfilling the inclusion criteria were invited to participate in the survey. A subgroup responded and participated in the study. n = number of patients

The user expectations were assessed by measuring the subjective expectations of use at the time of indication. The subjective expectations encompassed questions on the importance of usefulness in general and 13 different use options of the robotic arm (Fig. 1). The weighting of the use options was determined by the Numeric Rating Scale (NRS). The weighting of defined usage options was classified into four groups: not important (0 points), somewhat important (1–3 points), important (4–6 points), and very important (7–10 points).

#### Causes of failed provision and duration of procurement process

After assessing the reasons for failed provision, a shortlist was compiled, including rejection by health insurance, patient refusal, and patient death before provision. The duration of time between medical indication for a robotic arm provision and the delivery (latency of provision) or failed procurement (latency of failed procurement) were assessed on the APST platform.

#### Frequency of use

The frequency of use was evaluated by categorizing it into the following groups: (1) several times per day (day and night times); (2) several times a day (only during daytime); (3) several times a week; (4) once a week; and (5) less than once a week. Additionally, the date of initial use was recorded.

#### User experiences

The user experience was assessed by measuring the subjective use experience after successful provision with the robotic arm. User experience was assessed by the NRS and classified into four groups: not important (0 points), somewhat important (1–3 points), important (4–6 points), and very important (7–10 points).

In addition, the overall importance and usefulness of the robotic arm, the feeling of safety when using the robotic arm, and the satisfaction with its reliability were determined after the assistive technology device was successful fitted. The weighting of these user experience was assessed by the NRS.

#### Recommendation of a robotic arm

Global satisfaction with the provision of a robotic arm was assessed by a score of the likelihood of recommendation (Net Promoter Score, NPS). By using the NPS the participants were asked the following question: "How likely is it that you would recommend the robotic arm to a friend or colleague with deficits in arm function?” The answers were given on a nominal scale of between 0 (extremely unlikely to recommend) and 10 (highly likely to recommend) points. The evaluation was performed according to the following system [21, 22]: likely recommendation (10 or 9 points, "promoters"), indifferent recommendation (8 or 7 points, "indifferent"), and unlikely recommendation (6–0 points, "detractors").

The NPS for satisfaction with the use of robotic arm was calculated by subtracting the percentage of patients who were “detractors" from the percentage of patients who were "promoters". The NPS is calculated as follows:

$$NPS \, = \, {\text{``}}promoters{\text{''}} \, \left( {in \, \% \, of \, all \, respondents} \right) \, minus \, {\text{``}}detractors{\text{''}} \, \left( {in \, \% \, of \, all \, respondents} \right).$$

The value range of the NPS is thus between plus ( +) 100 and minus ( − ) 100. An NPS with a positive value (greater than zero) is considered a supportive recommendation [21]. An NPS of + 50 is considered excellent [22].

### Data source

Data on the provision were assessed by the APST platform and provided for the APST Registry Study. Data on functional deficits, user expectations, usage frequency, and experiences were collected by the physician or study coordinator via a data sheet (demographic data, user expectations and experience, and the likelihood of recommendation).

### Statistical methods

Descriptive statistics (frequency in percent, mean, median, standard deviation in ± , and ranges) were used for the statistical analysis. Differences in frequencies between the two groups were assessed by Fisher´s exact test or Chi-square test and between-metric data by t-test, as appropriate. P values were reported at a 95% confidence interval. The data were analyzed using SPSS (version 27.0).

## Results

### Participants

Within the observation period, 158 patients were identified for whom APST case management initiated the provision of a robotic arm. All of these patients were invited to participate in the study. Ultimately, 85 patients were included in the expectation (Baseline) survey and analyzed. Of those included in the study, 32 patients (37.6%) were provided with a robotic arm within the observation period. Fourteen patients (43.8% of those provided) participated in the user experience survey (Follow-up survey; Fig. 1).

### Demographic and clinical characteristics

An overview of the demographic and clinical characteristics is provided in Table 1.

More male participants are presented in the cohort (69%, n = 59). However, gender differences within those cohorts were not significant (data not shown). The mean age at time of indication for the robotic arm was 53.1 years. Patients with the device were slightly younger than those not provided, but not significantly (53.1 vs. 57.6 years, *p* = 0.140). ALS progression rate in the cohort provided with the robotic arm was 0.74, whereas the progression rate in the cohort not provided was 0.58 (*p* = 0.459; Table 1).

### Functional deficits of the arms

All patients (n = 85) showed a loss of arm function at the time of indication of the robotic arm. The mean ALS subscore for arm function was 1.7 (SD: 2.0, range: 0–9). The classified severity of arm function is shown in Table 1.

### User expectations

**Fig. 2:**
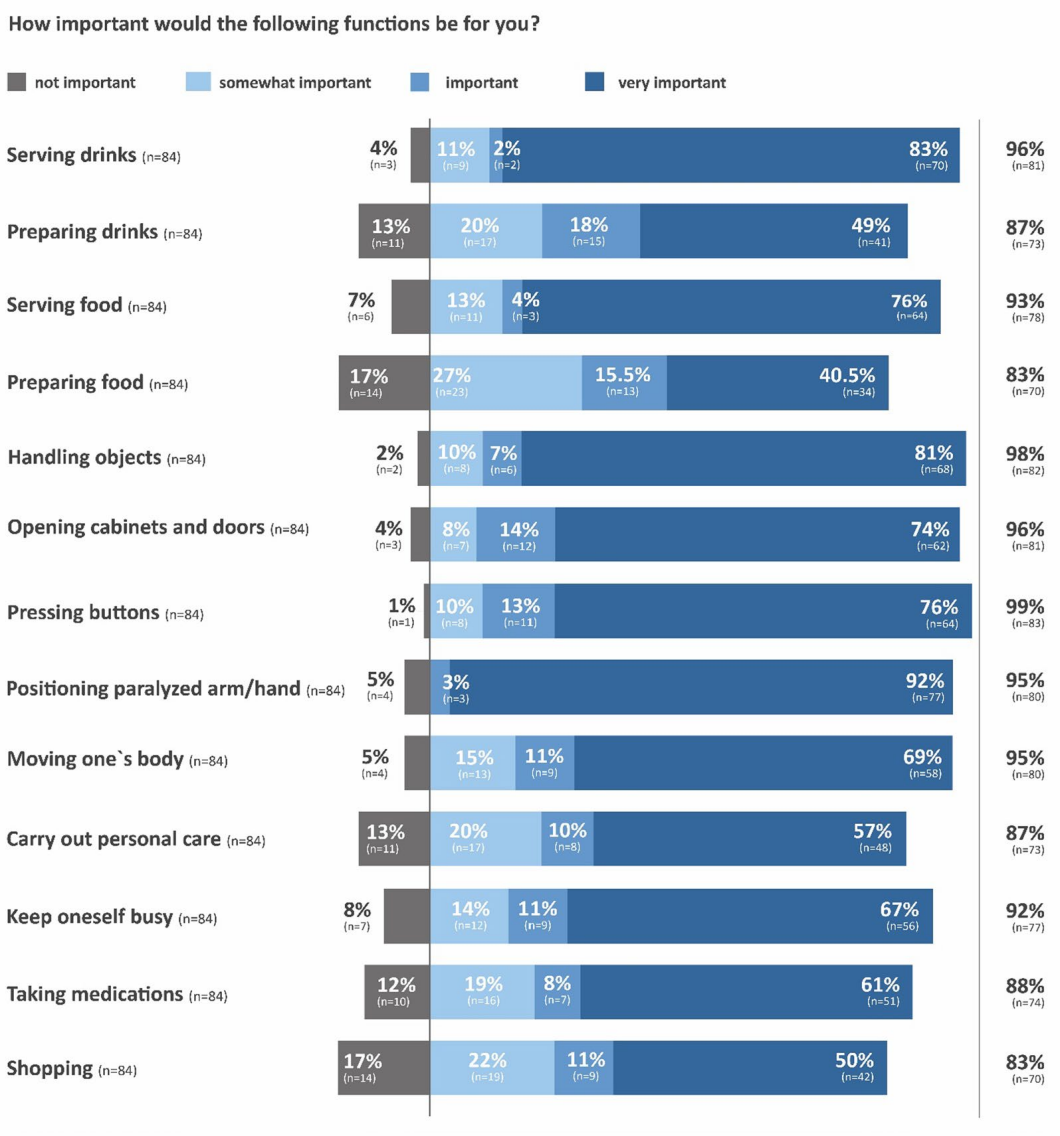
Weighting of the expectation of the treatment options of the robotic arm. The weighting of the treatment options was recorded by the Numeric Rating Scale (NRS; 0–10 points): not important (0 points), somewhat important (1–3 points), important (4–6 points), very important (7–10 points). n = number of patients

All patients rated the treatment option of a robotic arm as important. More than 80% of the patients expect the use case of handling objects for movements close to the body (such as scratching or putting on glasses), for pressing buttons, serving drinks, and opening cabinets and doors. Other uses include serving food, keep oneself busy (e.g., playing cards), personal care (e.g., applying makeup, shaving), positioning of paralyzed arm/hand, shopping, taking medications, and serving drinks and food (Fig. 2). Other use cases included tucking in or out of bed, operating a computer, turning pages, petting animals, telephoning, and watering flowers. The weighting of importance of the different use options is shown in Fig. 2.

### Causes of failed provision and duration of procurement process

The failure rate of the robotic arm analyzed was 62.4% (n = 53). For most patients, the reasons for not receiving a robotic arm were the unsuitability of the device (30.2%, n = 16, e.g., disease progression, lack of technical requirements) followed by the rejection by the health care insurance (28.3%, n = 15). 20.8% (n = 11) of the patients refused a fitting despite the indication, and 20.8% (n = 11) of the patients died before the provision. The latencies of provision and of failed procurement are shown in Table 2.

**Table 2:** Latency in the procurement process

Characteristics	Description	Latency, days, mean (SD, R)
Latency of provision	Total	229.5 (140.6, 78–645)
Latency of failed procurement	Total	196.7 (157.3, 7–588)
	Due to rejection by health insurance	208.5 (162.7, 50–560)
	Due to unsuitability of the device	188.8 (171.5, 7–503)
	Due to refusal by patient	214.7 (180.3, 14–588)
	Due to patients’ deaths before the provision	174.1 (116.9, 53–463)

Latency of provision of the robotic arm is defined as time interval (in days) between indication and procurement of the device. Latency of failed provision of the robotic arm is defined as time interval (in days) between indication and failed procurement of the device. Abbreviations: n = number of participants; SD = standard deviation; R = range

### Frequency of use

All patients provided with a robotic arm received it montaged in a wheelchair (100%, n = 14). Most patients used the device daily (71.4%, n = 10), and 28.6% (n = 4) several times a week.

### User experiences

**Fig. 3:**
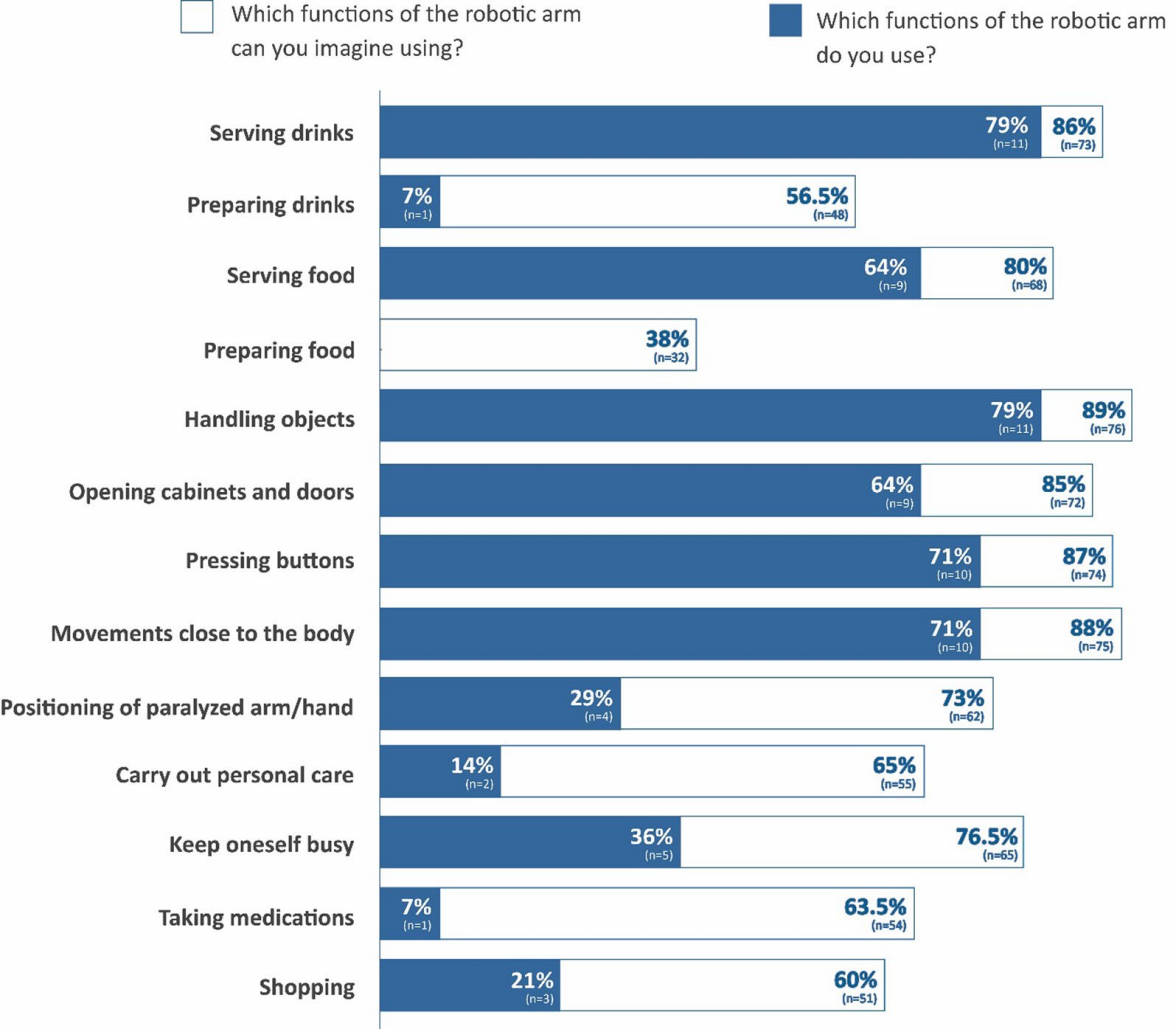
Expectation of treatment options vs. experience with using the functions of the robotic arm. The answers of the expectations of the treatment options followed the question "Which functions of the robotic arm can you imagine to use?" The answers of the experience followed the question "Which functions of the robotic arm do you use?"

Over 70% used the robotic arm for handling objects, serving drinks, to perform near-body movements (such as scratching or putting on glasses), and push buttons. Other use cases included serving food and opening doors and cabinets. Furthermore, patients used the robotic arm for keep them busy, for positioning their paralyzed arms, practice personal hygiene, prepare drinks and take medication. None of the patients used the robotic arm to prepare meals (Fig. 3).

**Fig. 4:**
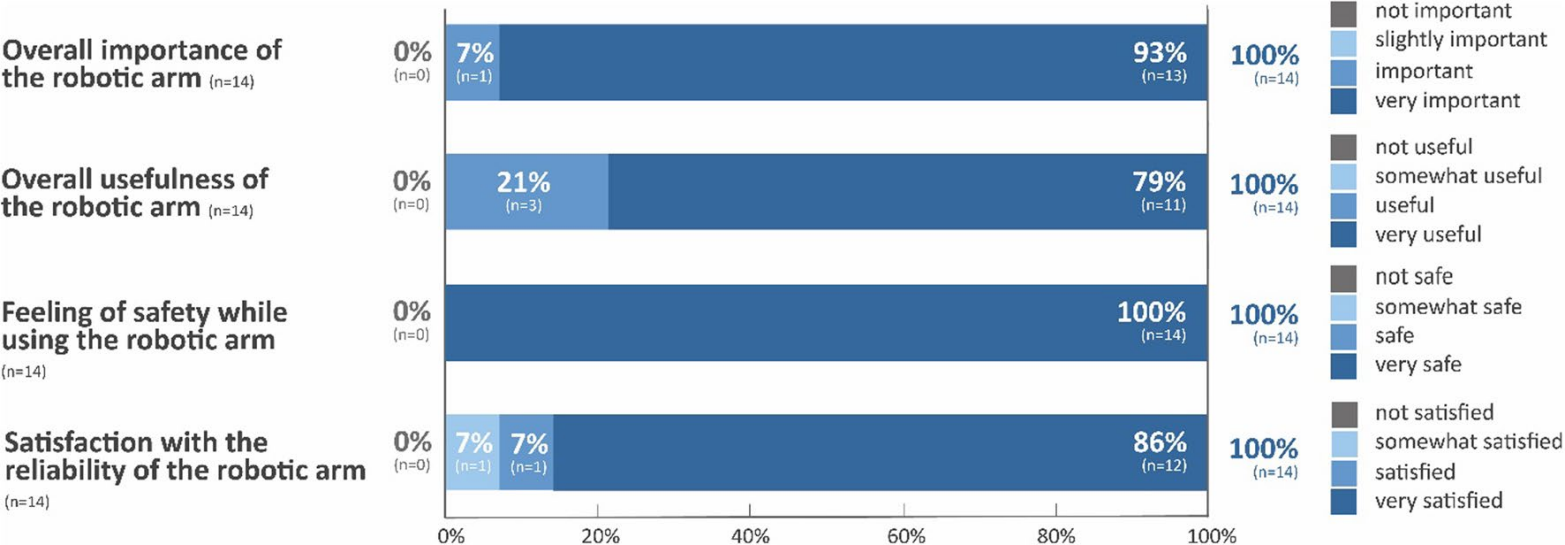
Overall usefulness of the robotic arm. The importance of the robotic arm was assessed by the Numeric Rating Scale (NRS; 0–10 points): not important (0 points), slightly important (1–3 points), important (4–6 points), very important (7–10 points). The usefulness of the robotic arm was assessed by the Numeric Rating Scale (NRS; 0–10 points): not useful (0 points), somewhat useful (1–3 points), useful (4–6 points), very useful (7–10 points). n = number of patients. The feeling of safety while using the robotic arm was recorded by the Numeric Rating Scale (NRS; 0–10 points): not safe (0 points), somewhat safe (1–3 points), safe (4–6 points), very safe (7–10 points). Satisfaction with the reliability of the robotic arm was measured by the Numeric Rating Scale (NRS; 0–10 points): not satisfied with the reliability (0 points), somewhat satisfied with the reliability (1–3 points), satisfied with the reliability (4–6 points), very satisfied with the reliability (7–10 points). n = number of patients

All provided patients (100%) rated the robotic arm as important and useful, felt safe while using the assistive device, and were satisfied with the reliability of the product (Fig. 4).

### Recommendation for the robotic arm

**Fig. 5:**
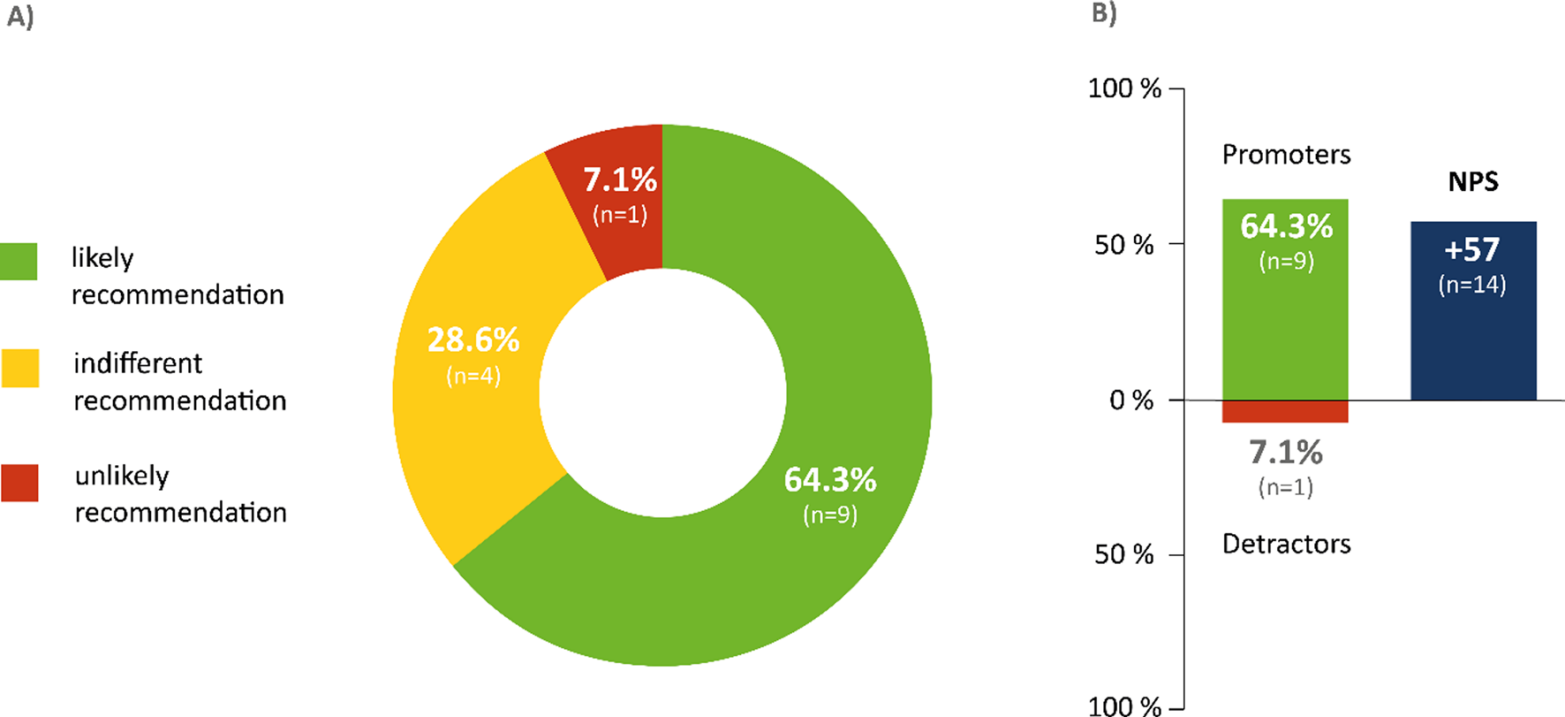
Recommendation of robotic arm using the Net Promoter Score (NPS). The NPS was applied to assess the patients’ likelihood of recommending this assistive technology device. This metric was calculated based on responses to a question: “How likely is it that you would recommend the robotic arm to a friend or colleague who suffers from ALS?” The answer was rated between 0 points (absolutely unlikely recommendation) and 10 points (highest likelihood of recommendation). Patients who responded with a score of 9–10 were considered as “promoters”. Those who rated the medication with 7 or 8 were classified as “indifferent”. The patients who responded with 6–0 points were defined as “detractors”. The NPS is calculated by subtracting the percentage of patients who are detractors from the percentage of patients who are promoters. n = number of patients

Of the patient cohort who participated in the user experience survey (n = 14), 64% strongly recommended the robotic arm, 29% showed an indifferent attitude, and only 7% of the patients did not recommend the product (0–6 points). The NPS was + 57 (Fig. 5).

## Discussion

ALS is a disease that causes progressive motor deficits. Unfortunately, despite of gene-based therapies in a subgroup of ALS-patients, there is currently no effective pharmacological therapy available for it. Therefore, substituting lost functions is crucial. Several established substitutions exist, such as percutaneous endoscopic gastrostomy (PEG) for swallowing function and ventilation therapy for reduced ventilatory function. Complex wheelchairs and other mobility aids can also be substituted for reduced leg and trunk function.

In this context, the robotic arm can be considered a substitution for lost arm functions. A small group of ALS patients has already been provided with robotic arms, but limited data is available to date, on expectations and actual experiences with this treatment option. Robotic arm systems used in clinical practice are complex and expensive, so it is important to assess the expectations of use cases and experiences in this treatment option. This observational study is the first systematic survey of clinical practice data on the provision and use of robotic arms for ALS patients.

### Sample selection

In this study, use cases for a robotic arm and experiences with the provision of the device were analyzed in patients with ALS at nine specialized ALS centers in Germany collaborating on managed care for ATD [4]. Systematic assessment of “real-world expectations and experiences” was facilitated using a digital management and research platform called Ambulanzpartner [2, 4, 13–16, 23]. Currently, more than 1,900 patients with ALS participate in the care management and the research projects of Ambulanzpartner representing approximately about 25% of the ALS population in Germany [24]. Digitalization of procurement processes allowed the systematic analysis of the multi-step robotic arm procurement process and the real-world expectations and experiences with this complex and highly personalized ATD. Remarkably, this approach allowed 85 patients to be included in the user expectation survey. Despite the advantages of the platform-based registry, the findings of this study must be considered in the context of their limitations. All participating study sites were specialized centers for ALS. Thus, it may be possible that patient characteristics in this study resulting from the procurement process, user expectations and experiences might deviate outside dedicated ALS centers.

Furthermore, considering that the indication for the robotic arm is only given to a limited number of patients with ALS, the study cohort covered a relatively small sample size. Not all patients with arm paresis are suitable for a robotic arm provision, as patients must also be able to operate and control the device. The proportion of patients provided—compared to the prevalence of ALS and the frequency of arm paresis—is considerably low and therefore, an underuse can be assumed. Furthermore, insufficient knowledge and communication of the potential of robotic assistance systems in ALS should be considered. Given the limited sample-size, the statistical analysis was therefore confined to descriptive methods and reduced the power of the study. A subgroup of patients was not provided with the robotic arm (62%), although they had a medical indication for this device. The rejection rates and latency of provision (197 days) of the robotic arm is higher than for other complex and highly individualized ATD´s in ALS [4]. As the robotic arm is a complex and cost-intensive device that exceeds the usual costs of a complex wheelchair, a longer health insurance review process (208 days) and high rejection rate were to be expected. Even though it is known that the indication for costly ATD´s is reviewed more frequently refused by health care insurances than less expensive devices, the rejection rate by the health insurance for the robotic arm was low in comparison [4]. Other reasons for the non-provision were based on patient-related factors. Long latencies in the provision process were one main reason why the device is no longer suitable as the disease progresses (189 days) or patients died before provision (174 days). In addition, delays in obtaining an electric powered wheelchair can lead to an even more significant delay in providing a robotic arm, which is necessary for using the arm. The interpretation of this data is limited because the reasons for non-provision—especially the patient related factors, and the latency in the provision were not explored systematically in this study.

Moreover, the results from the user experience survey should be interpreted with caution in light of the specific reasons for loss to follow-up. Specific reasons for loss to follow-up, e.g. patients dropping out because they are too severely affected to return to the study center or patients dying before the survey, are known to be methodological limitations in serious diseases surveys [25]. In this study, those losses to follow-up were favored due to latency in the data collection process, as the satisfaction survey was scheduled three months after provision of the robotic arm. Methods such as remote collection of patient-reported outcomes (e.g. via the “ALS-App”; Additional file 1) may be used in future studies to reduce high loss to follow-up rates.

### User expectations

All patients rated the treatment option of a robotic arm as important. The expectation of self-determined performance dominates. Remarkably, performing tasks on one's own body (e.g. scratching, wiping off excess saliva or putting on glasses) indicates that autonomy and gain in privacy are most relevant. The positive expectation of use—indicating the high importance of the care option—underlines the care potential of an arm robot in patients with a high-grade deficit of the motor arm functions.

### User experiences

The evaluation of user experiences showed overall a high level of usefulness and satisfaction with the robotic arm. Most ALS patients (71%) used the robotic arm several times a day, thus implying a high level of acceptance of the device [26]. Thus, in people with ALS, the use of the robotic arm enabled the patient to compensate for losses of major functions, especially independent drinking and handling of objects, pressing buttons and opening doors, as well as body-related movements [2]. The satisfaction with the reliability and the perception of safety were very high. Given the low case numbers, differences in user experiences between different patient groups (e.g., between different age groups or genders) could not be analyzed.

### Recommendation of robotic arm by patients

The NPS served as a robust instrument for assessing products and services but is still limited in evaluating medical products and services. Although the validation of this score in medicine is still limited, the NPS finds growing use in outcome research, mainly due to the simplicity of the method and the established calculation matrix [27, 28] and is increasingly applied in medical research [16, 23, 29]. NPS results > 50 are considered “excellent”. This result suggests high treatment satisfaction of ALS patients with the investigated robotic arm. However, due to limited experience with this score in the medical setting, caution is warranted when transferring the NPS system of validating products and services to treatment options. Given the low case numbers, differences in NPS between different patient groups (e.g., between different age groups or genders) could not be analyzed. Moreover, due to loss to follow-up, 18 patients could not be invited to take part in this survey. Therefore, it needs to be considered that the recommendation could have been different if full participation had been achieved. As NPS data on robotic arms have not yet been published in ALS, and there are no comparative data for other robotic assistance systems, the obtained NPS score for robotic arms is, therefore, to be regarded as a baseline for further studies.

## Conclusion

In conclusion, this observational study demonstrated frequent use of robotic arm systems, implying a high acceptance of the device. The results showed positive user experience and high satisfaction, which underline the potential of the robotic arm in ALS. The robotic arm allows patients to experience independence in daily activities. The positive user experience and high satisfaction with the provision and use underscore the potential of robotic arms in ALS. Increased knowledge and education about the device’s potential in ALS is needed to increase awareness and procurement of robotic arms in ALS. In future studies, it would be interesting to investigate how robotic arms improve the function of daily living and, thus, the quality of life of people living with ALS.

## Supplementary Information

Additional file 1

## Data Availability

The data that support the findings of this study are available from the corresponding author upon reasonable request.
