# Supplementary material for: User expectations and experiences of an assistive robotic arm in amyotrophic lateral sclerosis: a multicenter observational study
Source: Neurol Res Pract. 2024 Aug 23;6(1):42. doi: 10.1186/s42466-024-00342-3 (PMC11344397; doi:10.1186/s42466-024-00342-3)
Supplement: Supplementary file 1 — Additional file 1 [file 42466_2024_342_MOESM1_ESM.pdf]

Additional file 1: Links to mobile app for digital assessment of ALSFRS-R and patient-reported outcomes

The “ALS-App” is a digital health application for people with amyotrophic lateral sclerosis (ALS). The ALS-App makes it possible to record the individual progression of ALS by answering the ALSFRS-R and participating in surveys to record patient-reported outcomes. Patients can download the “ALS-App” via the App Store (for iOS) or Google Play (for Android).

Apple Store: https://apps.apple.com/us/app/als-app/id1468079806?l=de&ls=1

Google Play: https://play.google.com/store/apps/details?id=de.ambulanzpartner.alsapp
